# Supplementary material for: Digital cultural intelligence and its role in enhancing expatriate work adjustment: A configurational approach in global work environments
Source: PLoS One. 2026 Feb 13;21(2):e0342645. doi: 10.1371/journal.pone.0342645 (PMC12904368; doi:10.1371/journal.pone.0342645)
Supplement: S3 File — (PDF) [file pone.0342645.s003.pdf]

# Digital Cultural Intelligence and Expatriate Work Adjustment Survey

This questionnaire is part of the research study titled: "Digital Cultural Intelligence and Its Role in Enhancing Expatriate Work Adjustment: A Configurational Approach in Global Work Environments."

Please read each statement carefully and indicate your level of agreement using the following scale:
1 = Strongly Disagree | 2 = Disagree | 3 = Neutral | 4 = Agree | 5 = Strongly Agree.

## Section One: Demographic Data

1. Age: [ ] Under 30 [ ] Above 30

2. Gender: [ ] Male [ ] Female

3. Educational Level: [ ] Bachelor's degree [ ] Master's degree [ ] Doctorate

4. Years of International Experience: [ ] Less than 10 years [ ] More than 10 years

5. Type of Job or Sector: [ ] Healthcare [ ] Education [ ] Banking [ ] Tourism

### Metacognitive CQ

| Item | 1 | 2 | 3 | 4 | 5 |
|---|---|---|---|---|---|
| I review my strategies before initiating any digital communication with people from different cultures. | | | | | |
| I consciously think about cultural differences during digital meetings. | | | | | |
| I learn from my past experiences to enhance my digital communication with people from various cultures. | | | | | |
| I adjust my way of thinking when encountering new situations in multicultural digital environments. | | | | | |

### Cognitive CQ

| Item | 1 | 2 | 3 | 4 | 5 |
|---|---|---|---|---|---|
| I am knowledgeable about the digital communication customs and traditions of other countries. | | | | | |
| I understand different digital protocols for dealing with global cultures. | | | | | |
| I know how to use various digital tools to communicate effectively with people from diverse cultural backgrounds. | | | | | |
| I recognize the importance of time and meeting management in virtual meetings across cultures. | | | | | |

| | | | | | |
|---|---|---|---|---|---|
| I can explain the differences between digital communication styles across countries. | | | | | |

## Motivational CQ

| Item | 1 | 2 | 3 | 4 | 5 |
|---|---|---|---|---|---|
| I enjoy using digital tools to communicate with individuals from different cultures. | | | | | |
| I feel enthusiastic about developing my digital skills to better understand other cultures. | | | | | |
| I put in extra effort to engage with others through digital platforms despite cultural challenges. | | | | | |
| I see interacting with other cultures as an opportunity to expand my professional and personal experience. | | | | | |

## Behavioral CQ

| Item | 1 | 2 | 3 | 4 | 5 |
|---|---|---|---|---|---|
| I adjust my tone and behavior during digital meetings to match the other culture. | | | | | |
| I use culturally appropriate digital expressions (such as emojis or comments). | | | | | |
| I ensure that I choose the appropriate digital language for each culture during conversations. | | | | | |
| I modify my writing or speaking style in digital communication based on others' cultural norms. | | | | | |

## Work Adjustment

| Item | 1 | 2 | 3 | 4 | 5 |
|---|---|---|---|---|---|
| I feel comfortable performing my job duties in the new work environment. | | | | | |
| I am able to understand work policies and procedures easily. | | | | | |
| I find that the work environment supports me in performing my tasks efficiently. | | | | | |

## Interaction Adjustment

| Item | 1 | 2 | 3 | 4 | 5 |
|---|---|---|---|---|---|
| I build good relationships with colleagues from different cultural backgrounds. | | | | | |
| I find it easy to communicate with supervisors and management in the new workplace. | | | | | |
| I enjoy participating in social activities at work. | | | | | |

## General Adjustment

| Item | 1 | 2 | 3 | 4 | 5 |
|---|---|---|---|---|---|
| I feel comfortable dealing with the lifestyle in the host country. | | | | | |
| I adapt easily to local laws and customs. | | | | | |
| I can adjust to daily living conditions outside the workplace. | | | | | |

## Perceived Organizational Support

| Item | 1 | 2 | 3 | 4 | 5 |
|---|---|---|---|---|---|
| I feel that the organization cares about my well-being and strives to facilitate my adjustment at work. | | | | | |
| The organization provides me with the resources I need to perform my job effectively. | | | | | |
| I receive technical and professional support when needed. | | | | | |

## International Experience

| Item | 1 | 2 | 3 | 4 | 5 |
|---|---|---|---|---|---|
| I have prior experience working in international or multicultural environments. | | | | | |
| I have previously participated in international training programs or conferences. | | | | | |
| I believe my previous experience helps me adapt more quickly. | | | | | |

## Language Proficiency

| Item | 1 | 2 | 3 | 4 | 5 |
|---|---|---|---|---|---|
| I can communicate in the host country's language without significant difficulties. | | | | | |
| I use the local language effectively in daily conversations. | | | | | |
| I can understand the professional terminology used in my work environment. | | | | | |

## Digital and Cultural Training

| Item | 1 | 2 | 3 | 4 | 5 |
|---|---|---|---|---|---|
| I have received digital training to understand communication styles in multicultural environments. | | | | | |
| I feel that the training I received helped me improve my interactions with colleagues from different cultures. | | | | | |
| I have sufficient knowledge of digital tools that enhance international communication. | | | | | |
