# Supplementary material for: Digital cultural intelligence and its role in enhancing expatriate work adjustment: A configurational approach in global work environments
Source: PLoS One. 2026 Feb 13;21(2):e0342645. doi: 10.1371/journal.pone.0342645 (PMC12904368; doi:10.1371/journal.pone.0342645)
Supplement: Attachment — Submitted filename: Response_to_Reviewers.docx.docx [file pone.0342645.s004.docx]

***The first reviewer’s Comment***

***Comment 1:*** *The manuscript does not sufficiently explain why the GCC context is theoretically and empirically important, given the uniqueness of GCC labor markets.*

***Response:*** We sincerely thank the reviewer for highlighting the need for a stronger contextual grounding of the Gulf Cooperation Council (GCC) setting. We fully agree that the GCC labor market possesses distinctive institutional, demographic, and regulatory characteristics that warrant explicit theoretical engagement rather than being treated merely as an empirical backdrop.

In response, we have substantially revised the Introduction to include a dedicated contextual subsection that theorizes the GCC as a generative research context for expatriate work adjustment and digital cultural intelligence. Specifically, we now elaborate on (a) the structurally expatriate-dependent nature of GCC labor markets, (b) the temporariness and regulatory embeddedness of expatriate employment, and (c) the coexistence of rapid digital transformation with strong institutional and cultural boundaries. These features collectively intensify the salience of digital–cultural fit, rendering the GCC context theoretically distinctive for configurational HRM research.

This revision clarifies why findings derived from the GCC are not merely context-bound but instead offer theoretically transferable insights into expatriate adjustment under highly institutionalized, digitally mediated global work environments.

**Location of revision:**

Introduction – newly added subsection on the GCC labor market context.

***Comment 2:*** *The reviewer suggests shifting the focus from “diversity of cultures” to heterogeneous adaptation pathways among expatriates from similar backgrounds and encourages engagement with segmented assimilation and social network theories.*

***Response*:** We sincerely thank the reviewer for this insightful and theoretically valuable suggestion. We fully agree that expatriate adjustment should not be conceptualized solely as a function of cross-cultural diversity, but rather as a process characterized by heterogeneous adaptation trajectories, even among expatriates with similar cultural or demographic backgrounds.

In response, we have refined the theoretical framing of the manuscript to explicitly emphasize intra-group heterogeneity in expatriate adjustment. Drawing on segmented assimilation theory and social network perspectives, we now argue that expatriates originating from similar cultural contexts may follow divergent adjustment pathways depending on their digital-cultural competencies, organizational embeddedness, and network positioning within the host environment. This refinement aligns closely with the configurational logic of the study and strengthens the theoretical justification for employing fsQCA to capture multiple equifinal paths to successful adjustment.

We have incorporated this perspective into the theoretical framework and discussion sections, thereby shifting the analytical emphasis from cultural difference per se to the interaction between individual capabilities, social embeddedness, and institutional context.

**Location of revision:**

Section 2 – Theoretical and Conceptual Framework

***Comment 3 :*** *The reviewer requests a more comprehensive justification of sample selection, particularly the choice of tourism, healthcare, and higher education sectors. The reviewer also asks whether descriptive evidence can be provided to demonstrate the representativeness of these sectors among expatriates, and whether fieldwork sites can be visualized at the city level while maintaining anonymity.*

***Response:*** We thank the reviewer for this constructive comment regarding sample selection and sectoral scope. We agree that clearer justification strengthens methodological transparency and interpretive rigor.

In response, we have expanded the Methodology section to provide a more systematic rationale for selecting tourism, healthcare, and higher education as the focal sectors. These sectors were deliberately chosen to capture structural and functional heterogeneity in expatriate work contexts within the GCC, particularly with respect to (a) degrees of service interaction, (b) knowledge intensity, (c) digital mediation of work, and (d) exposure to culturally embedded stakeholder interactions.

We have also added descriptive contextual statistics, drawing on regional labor market reports, to demonstrate the centrality of expatriate employment in these sectors across GCC economies. Rather than assuming homogeneity, we explicitly acknowledge and theorize sectoral differences in expatriate roles and adjustment demands, which further justify their inclusion within a configurational research design.

Regarding the visualization of fieldwork sites, we respectfully note that city-level mapping was constrained by ethical and confidentiality considerations. However, we have clarified the multi-city nature of data collection and the logic of geographic dispersion in the revised manuscript, while maintaining strict anonymity protections consistent with ethical research standards.

**Location of revision:**
Methodology – Sample Selection and Research Context.

***Comment 4****: The authors should present an introduction to fsQCA, both methodologically and conceptually.*

***Response*:** We thank the reviewer for this important suggestion. We agree that providing a clearer methodological and conceptual introduction to fuzzy-set Qualitative Comparative Analysis (fsQCA) enhances transparency and improves accessibility for readers who may be less familiar with configurational approaches.

In response, we have added a dedicated subsection in the Methodology section that introduces fsQCA both conceptually and methodologically. This subsection explains the core principles of fsQCA—namely configurational causation, equifinality, and causal asymmetry—and clarifies why fsQCA is particularly well suited for examining expatriate work adjustment in heterogeneous and institutionally complex contexts such as the GCC. We also contrast fsQCA with conventional regression-based approaches to highlight its analytical advantages for the present research objectives.

**Location of revision:**
Methodology – Analytical Strategy (new subsection introducing fsQCA).

***The second Reviewer***

***Comment1* :** *The authors should introduce the economic and policy contexts of Gulf Cooperation Council countries, as readers outside the Middle East may have limited familiarity with the region and its labor market.*

***Response:*** We thank the reviewer for highlighting the importance of contextualizing the GCC for an international readership. We fully agree that a clear explanation of the region’s economic and labor market characteristics is essential for situating the study.

In response, we have substantially expanded the Introduction to include a dedicated discussion of the GCC economic and policy context. The revised manuscript now explains the structural dependence of GCC labor markets on expatriate employment, the institutional features of contract-based and regulated mobility systems, and the distinctive policy environment shaped by national development visions across the region. We further clarify how these characteristics differentiate the GCC from traditional Western expatriation contexts and why they render the region theoretically significant for studying expatriate work adjustment and digital cultural intelligence.

This contextual addition is designed to orient readers unfamiliar with the Middle East while directly supporting the study’s theoretical framing and research design.

**Location of revision:**
Introduction – expanded subsection on the GCC economic and labor market context.

***Comment 2:*** *The reviewer notes that despite discussing “environment fit” and “cultural fit,” the manuscript does not sufficiently engage with the literature on social networks. The reviewer suggests incorporating prior empirical studies on cultural and demographic social networks and clarifying how this literature informs the study, given that the paper is empirical rather than purely theoretical.*

***Response:*** We thank the reviewer for this important observation and fully agree that the literature on social networks is highly relevant to understanding environment fit and cultural fit in expatriate contexts. In response, we have substantially strengthened the literature review by integrating key empirical studies on social networks, acculturational homophily, and relational embeddedness, and by explicitly linking these perspectives to expatriate work adjustment.

Specifically, we now draw on empirical research demonstrating how social and professional networks shape access to information, support, and opportunities for cultural learning, thereby influencing adjustment outcomes. Rather than treating social networks as an additional variable, we position them as a contextual mechanism through which digital cultural intelligence facilitates alignment between individuals and their work environments. This integration allows us to ground the conceptual discussion of environment fit and cultural fit in well-established empirical findings, while remaining consistent with the study’s configurational design.

These revisions ensure that the manuscript engages more directly with relevant empirical literature and clarifies how social network perspectives inform the interpretation of the results.

**Location of revision:**
Literature Review – expanded subsection on social networks and expatriate adjustment; Discussion section.

***Comment 3:*** *The reviewer notes several writing issues related to (1) the inconsistent use of abbreviations that are not clearly defined (e.g., EWA, HRM), and (2) the presentation style in later sections of the manuscript, where the writing shifts toward bullet-point formats rather than coherent academic prose.*

***Response:*** We thank the reviewer for this helpful comment regarding clarity and presentation. In response, we have undertaken a comprehensive editorial revision of the manuscript.

First, all abbreviations are now clearly defined at their first occurrence in the text (e.g., expatriate work adjustment [EWA], human resource management [HRM], digital cultural intelligence [DCI], and Gulf Cooperation Council [GCC]), and their usage has been standardized throughout the manuscript.

Second, we have revised Sections 4.5 through 5.5 to replace bullet-point-style presentations with fully developed academic paragraphs. These sections now present the results and discussion in a coherent narrative form, with improved transitions and clearer argumentative flow.

Finally, the manuscript has undergone careful language and style editing to ensure consistency, readability, and adherence to academic writing conventions.

**Location of revision:**
Throughout the manuscript; particularly Sections 4.5–5.5.

***Comment 4*:** *Since it is a qualitative paper, the reviewer suggests including records of interviews or participants’ original words to enrich the presentation of findings.*

***Response:*** We thank the reviewer for this thoughtful suggestion and appreciate the interest in gaining deeper insight into participants’ experiences. We would like to respectfully clarify that the present study is not a qualitative interview-based investigation, but rather a configurational empirical study employing fuzzy-set Qualitative Comparative Analysis (fsQCA) based on calibrated survey data.

fsQCA is a set-theoretic analytical approach that differs fundamentally from qualitative interview methods. While it adopts configurational logic, it relies on systematically collected quantitative indicators rather than narrative or conversational data. Consequently, the inclusion of interview excerpts or participants’ verbatim statements would not be methodologically consistent with the study design or data collection strategy.

To address potential ambiguity, we have clarified the methodological positioning of the study in the Methodology section, explicitly distinguishing fsQCA from qualitative interview-based research. We have also strengthened the interpretive discussion of the configurational results to provide richer, theoretically grounded explanations of expatriate experiences without implying qualitative data collection.

**Location of revision:**
Methodology – clarification of research design and analytical approach.

***Comment 5*:** *The reviewer expresses hesitation in framing the manuscript as a “global HR” paper, noting the uniqueness of the GCC context. The reviewer suggests that unless a comparative design is adopted, the authors should better articulate why the GCC context is globally relevant, emphasizing theoretical rather than practical generalization.*

***Response:*** We thank the reviewer for this thoughtful and constructive comment. We agree that the GCC represents a highly distinctive institutional and labor market context, and that the study does not aim to offer direct practical generalizations across all global HR settings.

In response, we have refined the framing of the manuscript to clarify that its contribution lies primarily in theoretical generalization rather than universal practical applicability. Specifically, we now position the GCC as a theoretically generative context through which broader insights into expatriate adjustment, digital cultural intelligence, and networked forms of environment fit can be developed. Rather than presenting the study as a comparative global HR analysis, we explicitly emphasize that the findings contribute to international HRM theory by illustrating how adjustment processes unfold under conditions of strong institutional constraint, high expatriate dependence, and digital mediation.

We have revised the Introduction and Discussion sections to articulate why the GCC context is analytically valuable for global audiences, while acknowledging its contextual specificity. This reframing ensures conceptual clarity and aligns the manuscript with the reviewer’s recommendation.

**Location of revision:**
Introduction (theoretical positioning); Discussion (theoretical implications).
